# Reconfigurable hardware-accelerated, multi-channel, adaptive temperature control platform of VCSELs for high-density fNIRS/DOT

**DOI:** 10.1364/BOE.562181

**Published:** 2025-06-03

**Authors:** Qiao He, Yunjia Xia, Xuhao Zhang, Xinkai Zhou, Yu Liu, Yixuan Huang, Xiangyu Zhou, Aobo Ren, Hubin Zhao, Jiang Wu

**Affiliations:** 1 The Institute of Fundamental and Frontier Sciences, University of Electronic Science and Technology of China, Chengdu 611731, China; 2 The HUB of Intelligent Neuro-engineering (HUBIN), CREATe, Division of Surgery & Interventional Science, University College London, London WC1E 6BT, UK; 3 The Sonosilicon Co., Ltd, Hangzhou 310051, China

## Abstract

Functional near-infrared spectroscopy (fNIRS) and its advanced offshoot - diffuse optical tomography (DOT) are promising non-invasive neuroimaging techniques. The advancement of next-generation high-density fNIRS/DOT systems, particularly high-density wearable systems, requires compact light source arrays with high wavelength tuning precision and fine modulation capabilities. Vertical-cavity surface-emitting lasers (VCSELs) have emerged as a strong candidate for this purpose. However, VCSELs’ performance is highly sensitive to temperature variations, where heating effects induce wavelength shifts and output power fluctuations, leading to measurement drift and reduced accuracy in fNIRS/DOT data. Conventional multi-channel VCSEL temperature control methods face constraints due to limited computational resources and poor scalability. To address these limitations, we propose a reconfigurable hardware-accelerated temperature control platform based on the heterogeneous ZYNQ-7000 Field-programmable Gate Array (FPGA). By integrating a real-time proportional-integral-derivative (PID) algorithm into the programmable logic (PL), the platform achieves precise temperature regulation with an error margin of ±0.01 °C. Experimental validation demonstrates the encouraging capability of this proposed platform to regulate the temperature of over 100 VCSELs simultaneously while maintaining low resource utilization, ensuring efficient parallel control with large channel counts in real-time. The proposed reconfigurable architecture significantly enhances the reliability and scalability of VCSEL-driven fNIRS/DOT systems while maintaining sufficient resources for future implementations of extra functions. This platform not only improves the thermal stability of VCSELs-based wearable high-density fNIRS/DOT devices but also establishes a robust thermal-control framework for broader applications requiring high-density, thermally stable light source configurations.

## 1. Introduction

**Fig. 1.**
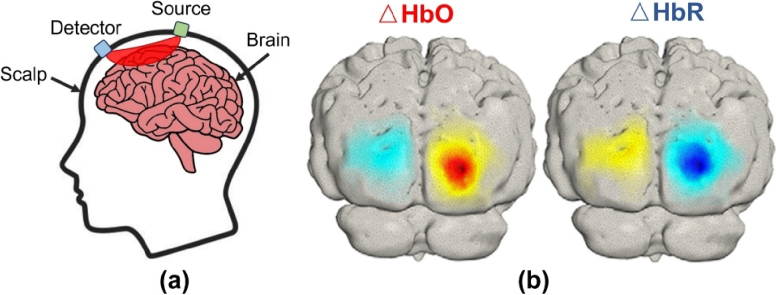
a) Illustration of the source-detector pair in an fNIRS system, depicting the light path; b) A 3D image illustrating the activation map obtained from visual stimulus using a DOT system.

Functional Near-Infrared Spectroscopy (fNIRS) is a non-invasive and wearable neuroimaging technique that measures brain activity by emitting near-infrared light through the skin and skull to the brain [1,2]. As shown in Fig. 1(a), a portion of this light is scattered back to the surface, carrying information about changes in the concentrations of oxygenated (HbO) and deoxygenated hemoglobin (HbR) in cortical regions, which are closely associated with local neuronal activity [1]. Diffuse Optical Tomography (DOT) extends the principles of fNIRS by employing a denser array of near-infrared light sources and detectors at varying separations [3]. This enables overlapping spatial sampling and the reconstruction of three-dimensional images of cortical hemodynamic activity (shown in Fig. 1(b)), offering improved spatial resolution and depth sensitivity compared to conventional fNIRS [4]. However, the spatial resolution of wearable DOT is partially constrained by the physical size of the light sources and detectors, highlighting the importance of compact designs of these optical components to achieve high-resolution imaging within the limited surface area of the scalp.

The commonly used fNIRS/DOT systems can be categorized into Continuous-Wave (CW), Frequency-Domain (FD), and Time-Domain (TD) systems. Among these, CW systems are the most widely employed, utilizing a constant-intensity light source such as light-emitting diodes (LEDs) or vertical-cavity surface-emitting lasers (VCSELs) to measure relative changes in hemoglobin concentration [3]. However, CW systems are inherently limited by their inability to differentiate between light absorption and scattering within tissue, thereby precluding the measurement of absolute hemoglobin concentration [5]. To address this limitation, advanced systems like FD and TD systems have been developed [5,6]. FD systems use modulated light sources to analyze phase shifts, allowing for photon pathlength estimation and absolute hemoglobin concentration measurement. TD systems, in contrast, emit ultrashort light pulses and detect the temporal distribution of scattered photons, enabling the absolute measurement of the hemoglobin concentration.

Both FD and TD systems impose specific requirements not only on the size of the light sources but also on their performance characteristics. In particular, these systems require light sources capable of high-frequency modulation (in the case of FD) or ultrashort pulse generation with a high repetition rate (in the case of TD) [5,6]. However, while LEDs are acceptable for CW-fNIRS/DOT, they are unsuitable for these advanced modalities due to their limited modulation bandwidth and pulse generation capabilities. In contrast, VCSELs are well-suited for CW, FD, and TD systems, making them a versatile choice for fNIRS/DOT applications. Their compact size (<10 mm), high wavelength tuning precision (∼0.01 nm), a broad tuning range (>10 nm), and fine modulation capabilities, have emerged as a promising solution for wearable fNIRS/DOT systems make them well-suited for precise, high-resolution measurements in CW, FD and TD fNIRS/DOT systems [5].

Despite the several advantages of VCSELs as light sources for all the modalities of fNIRS/DOT systems, VCSELs’ performance is sensitive to temperature variations. Specifically, temperature increases can lead to wavelength shifts of approximately 0.06–0.1 nm/°C, as well as reductions in output power and increases in threshold current [7,8]. These temperature-induced changes can introduce measurement drift, compromising the accuracy of fNIRS/DOT data [9]. To maintain stable performance, suitable temperature control for VCSELs is essential. Current approaches include thermoelectric coolers (TECs), heat sinks, radiators, fans, and liquid cooling systems [10–12]. Among these, TECs are widely used for their high precision, fast response, and reliability [13,14]. However, conventional TEC controllers often rely on microcontrollers (MCUs), digital signal processors (DSPs), or application-specific integrated circuits (ASICs) as their primary control units, each of which has notable limitations [15,16]. MCUs are cost-effective and energy-efficient but lack the computational capacity to handle high-speed control [17]. ASICs provide high-speed performance but are associated with high development costs and limited scalability, particularly for multi-channel (>16 channels) VCSEL temperature control, a common requirement in DOT systems [18]. DSPs offer strong high-speed performance and multitasking capabilities but lack the flexibility needed for broader system integration [19]. Most prior works based on MCUs, DSPs, or ASICs are limited in scalability due to constraints in processing speed, serial computation architecture, and I/O capabilities. Consequently, such systems typically support no more than 16 to 32 temperature control channels. For example, He et al. [15] and Zhang et al. [16] demonstrated laser temperature control systems based on MCU and DSP architectures, respectively, with channel counts below 32. Reviews by Adebisi et al. [17] and Alkhafaji et al. [18] further emphasize that while MCUs and ASICs may offer advantages in cost and simplicity, they are not well suited for large-scale parallel control due to fixed-function limitations and poor system integration flexibility.

Some field-programmable gate arrays (FPGAs) provide a superior alternative to DSPs and other conventional controllers due to their high-speed and multi-channel parallel processing capabilities, as well as their high flexibility for integrating additional functionalities beyond temperature control [20–22]. This flexibility makes FPGAs an encouraging choice for developing advanced control systems, such as those required for temperature control of VCSELs. For instance, Jiang et al. [14] implemented a multi-channel FPGA/DSP hybrid temperature control system that supports up to 32 TECs. Furthermore, Liu et al. [23] developed a high-performance time-to-digital converter (TDC) on a Kintex-7 FPGA that successfully supports 128 parallel channels, demonstrating the scalability of FPGA architectures in high-throughput scenarios.

Heterogeneous platforms, such as the Xilinx ZYNQ 7000 series [24], combine a processing system (PS), such as a CPU, and programmable logic (PL), enabling efficient distribution of computational workloads. High-speed, computationally intensive tasks can be assigned to the PL, while the PS manages sequential logic and floating-point operations [24]. This architecture allows developers to allocate tasks based on complexity and nature, making these platforms ideal for prototyping and implementing complex systems, including comprehensive fNIRS/DOT system control. By utilizing both the PS and PL, heterogeneous platforms excel not only in precise VCSEL temperature control but also in managing broader requirements like raw data processing and overall system operation.

Leveraging the high-speed, parallel processing capabilities and functional flexibility of the ZYNQ 7000 heterogeneous platform, herein, we propose a high-precision, scalable temperature control system for VCSELs that can be well suited for new-generation wearable CW, FD and TD fNIRS/DOT systems. The system integrates a hardware-accelerated Proportional-Integral-Derivative (PID) control module, achieving microsecond-level response times and ±0.01 °C accuracy. This design establishes a robust technological foundation for next-generation applications such as wearable high-density fNIRS/DOT, which demand high-density light sources with rapid temperature control. The contributions of this work are as follows:

1. Proposed a hardware-accelerated PID control algorithm on FPGA, enabling low-latency and high-precision temperature control (±0.01 °C) for VCSELs, ensuring wavelength stability and maintaining stable output power of VCSELs to minimize measurement drift in fNIRS/DOT systems.
2. Investigated a scalable FPGA-based architecture leveraging parallel processing to control the temperature of over 100 VCSELs, ensuring uniform thermal management and stable optical performance in high-density fNIRS/DOT systems.
3. Established a scalable and high-precision temperature control framework, facilitating the integration of VCSELs into wearable fNIRS/DOT systems by enabling compact, high-density light source arrays with minimal thermal drift.

## 2. Adaptive temperature control platform

### 2.1. System architecture of the proposed platform

**Fig. 2.**
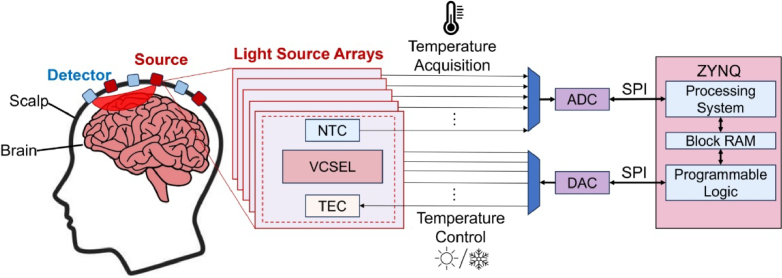
Architecture of the proposed ZYNQ-based temperature control system for VCSELs in fNIRS/DOT applications.

Figure 2 demonstrates the architecture of the proposed temperature control system for VCSELs. The temperature of each VCSEL within the light source array is continuously monitored by the negative temperature coefficient (NTC) thermistor, while thermal regulation is achieved via a TEC driver. The measured temperature from the NTC thermistor is transmitted to the ZYNQ-7000 SoC (XC7Z020, AMD) via the Serial Peripheral Interface (SPI) for real-time processing [25].

The ZYNQ-7000 SoC integrates PS and PL for efficient computation and control. The PS, equipped with dual ARM Cortex-A9 cores with floating-point units, converts the voltage across the NTC thermistor into resistance values, mapping them to the corresponding temperature. The PS receives the digitized voltage from the ADC and calculates the actual temperature using floating-point operations. The result, together with the target temperature, is stored in the shared Block RAM (BRAM), which can be accessed by the PL. The PL retrieves this data and runs a hardware-implemented PID algorithm to compute the required TEC control signal. This value is written back to the BRAM and read by the PS, which then updates the TEC driver via SPI. The PL, featuring 220 DSP units and 140 BRAMs (4.9 Mb total), executes real-time PID control, dynamically adjusting the TEC driver’s output voltage via SPI. The shared BRAM facilitates efficient data exchange between the PS and PL, minimizing latency and enhancing control precision. This closed-loop control system continuously stabilizes the VCSEL temperature at the predefined setpoint, ensuring reliable operation in high-density fNIRS/DOT applications.

### 2.2. Temperature acquisition module

The temperature of the VCSEL system is measured using the properties of an NTC thermistor. The resistance of NTC thermistors decreases as temperature increases [25]. The operating temperature of the VCSEL can be calculated using Eq. (1).

(1) $$R(T)=R_{r\text{ef}}\cdot e^{B\left(\frac{1}{T}-\frac{1}{T_{ref}}\right)}$$

Where R(T) is the resistance at temperature T , $R_{ref}$ is the resistance at the reference temperature $T_{ref}$ (298 K, 25 °C), and B is the thermal coefficient of the material (a constant determined by the material properties). Using a hardware voltage divider circuit, the resistance of the NTC thermistor is determined from the voltage measured by the analog-to-digital converter (ADC). By substituting the calculated resistance and the parameters from the VCSEL manual into Eq. (1), the operating temperature of the VCSEL is obtained.

The system employs the AD7124 chip (Analog Devices Inc.), a low-power, low-noise, fully integrated analog front-end device as the core component of the temperature acquisition circuit [26]. AD7124 is particularly well-suited for this application due to its high-precision 24-bit sigma-delta ADC, which offers configurable input options supporting up to four differential or seven single-ended/pseudo-differential channels. Furthermore, the inclusion of an on-chip programmable gain amplifier with adjustable gain settings ranging from 1 to 128 enables the direct measurement of low-amplitude signals. This capability is critical for accurately capturing small voltage variations generated by NTC thermistors, thereby enhancing the system's precision in temperature data acquisition.

**Fig. 3.**
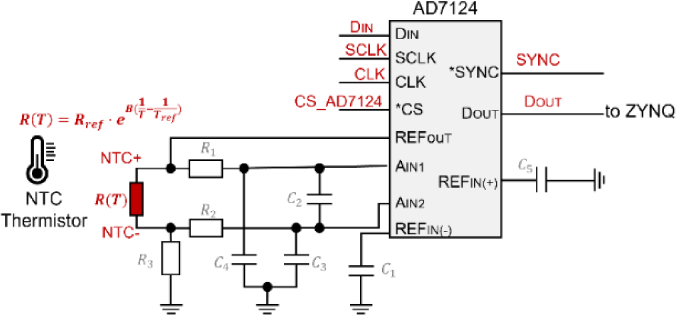
Schematic of the temperature acquisition circuit.

As shown in Fig. 3, to measure the resistance of the NTC thermistor, the ADC collects voltage values from a voltage divider circuit, where the thermistor is connected in series with a reference resistor (detection resistor). The reference resistor is delicately selected to match the nominal resistance of the NTC thermistor at a reference temperature of 298 K (25 °C). This configuration ensures that the output of the voltage divider is at the midpoint of the reference voltage, maximizing the dynamic range of the ADC and optimizing measurement accuracy. The high-resolution 24-bit ADC, combined with the programmable gain capability of the AD7124, allows the system to resolve small changes in resistance with high precision, which is essential for deriving accurate temperature readings from the thermistor. The AD7124 operates in differential mode with a ± 2.5 V input range and uses a 2.5 V internal reference. The sampling rate is configured to 100 samples per second per channel, balancing real-time responsiveness and noise performance. The programmable gain amplifier is set to a gain of 32 to amplify the millivolt-level voltage signals from the NTC thermistor. According to the AD7124 datasheet, under comparable low-noise configurations, the effective number of bits typically ranges from 17 to 18 bits. This resolution enables reliable sub-millidegree temperature discrimination, ensuring the 24-bit ADC’s precision is well utilized to meet the ±0.01 °C control accuracy required for VCSEL thermal stabilization. Additionally, the low-noise characteristics of the AD7124 minimize signal distortion, further enhancing the reliability of the measurements in this sensitive application.

### 2.3. TEC-based temperature control module

**Fig. 4.**
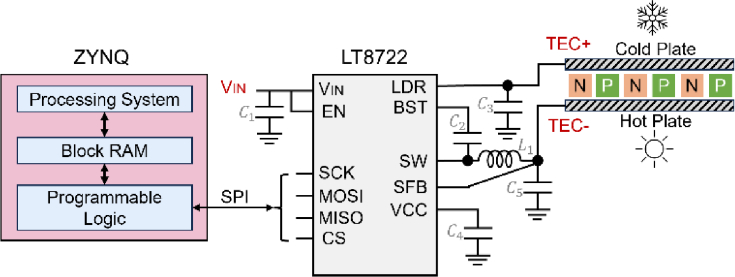
Schematic of the TEC driver circuit.

The TEC is critical in precisely adjusting and stabilizing the operating temperature of the VCSELs. By modulating the direction and magnitude of the current flowing through the TEC, the system can either cool or heat the VCSEL, ensuring stable operating conditions (Fig. 4). To achieve consistent and accurate temperature control over extended periods, the system incorporates the LT8722 chip (Analog Devices Inc.) as the core component of the TEC driving circuit [27]. The LT8722 is a high-performance, high-efficiency, monolithic full-bridge DC/DC converter specifically designed for driving TECs [27]. Its suitability for this application stems from several key features, including fast response times, high control precision, and a compact form factor. These characteristics contribute to the system's overall reliability and extended operational lifespan. The LT8722 is equipped with a 25-bit digital-to-analog converter (DAC) for precise control of the output voltage, as well as two 9-bit DACs for setting the range of positive and negative output current. This level of precision enables fine-grained control over the TEC's operation, ensuring optimal temperature regulation for the VCSEL. To enhance safety and reliability, the built-in protection features of LT8722—overcurrent protection and thermal shutdown—are enabled via SPI during system initialization. The output current limits for both sourcing and sinking directions are set to 4 A, consistent with the TEC module’s maximum ratings. These safeguards help prevent component damage under abnormal conditions, such as short circuits or thermal overload, thereby contributing to the long-term stability of the system.

The ZYNQ-7000 SoC communicates with the LT8722 via the SPI interface, enabling configuration of the pulse-width modulation signal's duty cycle and frequency. Through this interface, the system can adjust the output voltage and current ranges, as well as control the enable function of the chip. This adjustment regulates the direction and magnitude of the current across the TEC in real time, allowing the system to switch between cooling and heating modes as needed to maintain the VCSEL's operating temperature within the desired range.

### 2.4. PID module on PL

The high-precision temperature control system is based on the position PID algorithm, which achieves precise temperature regulation through proportional, integral, and derivative control [27]. The equation of PID control is expressed as:

(2) u(t)=Kp⋅
e(t)+Ki⋅
∫
0t⁡
e(τ
)dτ
+Kd⋅
de(t)dt

where u(t) is the control output; e(t) is the current error, defined as the difference between the target value and the actual value; $\int_{.0}^{t}e(\tau )d\tau$ is the accumulated error; $\frac{de(t)}{dt}$ is the rate of change of the error, approximated as e(t)−
e(t−
1)Δ
t , where e(t−
1) represents the previous error. $K_{p}$ , $K_{i}$ , and $K_{d}$ are the proportional, integral, and derivative gains, respectively [28].

The PID module is programmed on PL, which uses the voltage corresponding to the target temperature as a reference, computes the error between the target and actual temperatures, and calculates the next TEC drive voltage based on the predefined $K_{p}$ , $K_{i}$ , and $K_{d}$ coefficients. This iterative process ensures high-speed and precise control of the VCSEL’s operating temperature, maintaining stable performance.

### 2.5. Multi-channel temperature control

**Fig. 5.**
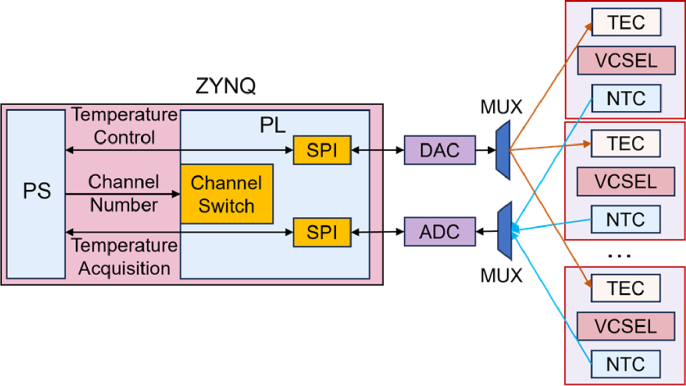
**System architecture for multi-channel VCSEL temperature control.** The PS generates configuration parameters for each VCSEL based on predefined requirements, which are then transmitted to the PL. The PL manages channel switching and SPI communication with DAC and ADC modules. The DAC controls the TECs for temperature control, while the ADC acquires temperature feedback from the NTC thermistors. A multiplexer (MUX) facilitates multi-channel selection, enabling precise calibration and data acquisition across multiple VCSEL channels.

The system is designed to support scalable multi-channel temperature control, enabling the management of up to 128 VCSELs. This scalability is achieved through a combination of hardware and software optimizations, leveraging the interaction between the PL and PS on ZYNQ [24]. As shown in Fig. 5, the PL is responsible for interfacing with the VCSELs, while the PS generates configuration parameters tailored to each VCSEL's requirements and distributes them to the SPI module and channel switching module on PL. This architecture ensures efficient calibration and data acquisition across multiple channels.

The PS generates configuration parameters for the ADC registers based on the specific requirements of each VCSEL. These parameters are transmitted to the PL's SPI interface. Simultaneously, the PS controls the channel selection module to activate the corresponding channel, enabling communication with the targeted VCSEL. The PS then initiates the SPI interface to read temperature-related data from the ADC chip for the currently active channel. The acquired data is processed to compute the temperature parameters for the corresponding VCSEL. The PS manages the channel switching module to sequentially or randomly activate the next channel, ensuring that all channels are periodically monitored and controlled. Based on the computed temperature parameters, the PS sends control signals to the SPI interface, which writes the necessary values to the DAC. This process adjusts the TEC drive voltage for the selected channel, ensuring precise temperature regulation for the corresponding VCSEL.

The system's ability to manage up to 128 channels is supported by several key design features. The PS employs a dynamic channel-switching algorithm, which alternates between channels at fixed intervals to ensure all channels are serviced within a defined timeframe. This approach maintains consistent temperature control across all VCSELs. Additionally, the PS and PL architecture separates responsibilities, with the PS handling parameter generation and computation, while the PL manages interface operations and data acquisition. This division of tasks optimizes resource allocation and enhances system scalability. By leveraging these design principles, the system can theoretically support up to 128 VCSELs without significant performance degradation, making it highly suitable for large-scale applications requiring robust and stable temperature control, such as high-density fNIRS/DOT.

To evaluate the temporal scalability of the proposed platform in high-channel-count applications, a theoretical timing analysis was conducted for a complete 128-channel temperature control cycle. This cycle includes SPI-based ADC data acquisition, hardware PID computation, and DAC register updates. The ADC read time per channel is approximately 3.2 µs, resulting in a total of ∼0.41 ms for all 128 channels. DAC configuration takes about 14.4 µs per channel, amounting to ∼1.84 ms in total. The PID computation, including a 512-point averaging step per channel, requires up to ∼209 ms for the full set of channels in the worst case. Data exchange between the PL and PS is conducted via shared BRAM, with per-access latency in the range of several clock cycles, which is negligible in the system context.

Summing these stages, the full 128-channel control cycle completes within ∼212 ms. This allows the system to operate at a control frequency of 4 Hz (i.e., one complete update every 250 ms), leaving a buffer of approximately 40 ms per cycle. This performance fully satisfies the temporal requirements of wearable fNIRS/DOT systems, which typically require temperature updates every 0.25 to 1 s.

## 3. Experimental results

### 3.1. Experimental setup

**Fig. 6.**
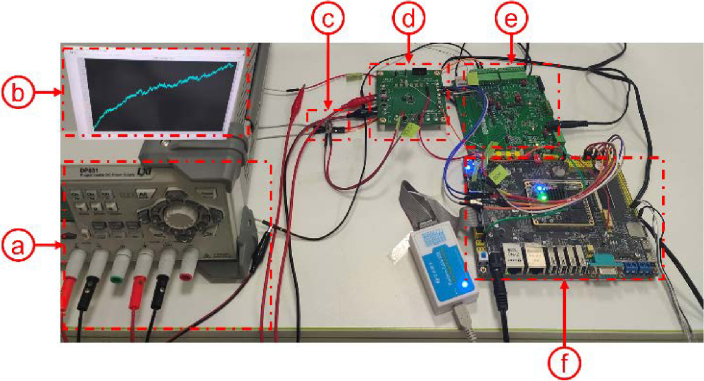
**Temperature control experimental system:** (a) power supply. (b) PC. (c) VCSEL integrated with NTC thermistor and TEC. (d) TEC driver. (e) temperature acquisition circuit. (f) ZYNQ.

Figure 6 illustrates the experimental setup for the temperature control system, which consists of the following key components: (a) A stable DC power source (b) An upper computer used for real-time acquisition of the temperature (c) VCSEL integrated with an NTC thermistor and TEC for temperature control. (d) The TEC Driver responsible for driving the TEC to regulate the VCSEL's temperature. (e) The temperature acquisition circuit which reads temperature-related voltage values and converts them for control operations. (f) The ZYNQ board serves as the main control module, coordinating temperature control through PS and PL.

**Fig. 7.**
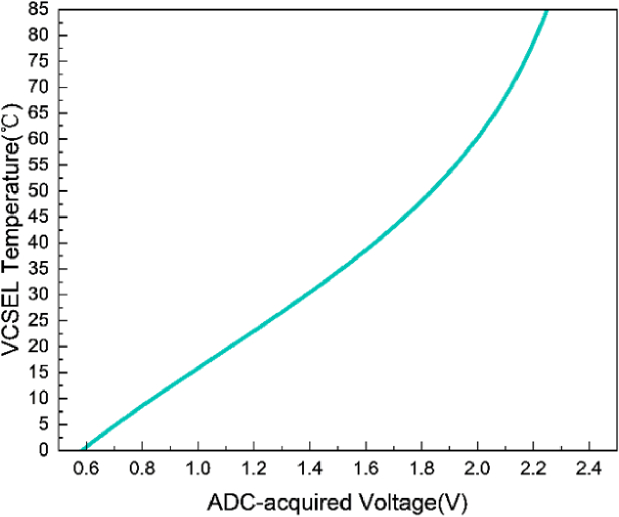
The relationship between the voltage acquired by the ADC and the real-time operating temperature of the VCSEL.

**Fig. 8.**
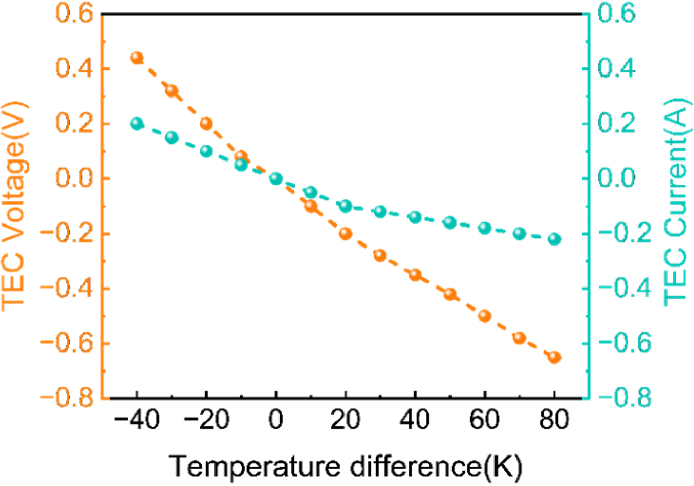
Output voltage and current of the LT8722 for TEC control under varying temperature differences.

A 761 nm VCSEL (Priolas) was used for performance evaluation, as this wavelength is in the common range of typical wavelengths that used in fNIRS/DOT system [29]. The parameters in Eq. (1) include thermal coefficient B of the NTC thermistor, which is specified as 3892 according to the datasheet, and the reference resistance $R_{ref}$ , which is 10 kΩ at a reference temperature $T_{ref}$ of 298 K (25 °C) [29]. To obtain the real-time resistance value of the NTC, a 10 kΩ precision resistor is connected in series with the NTC resistor, creating a voltage divider for the 2.5 V constant voltage source. Figure 7 illustrates the relationship between the voltage values acquired by the ADC and the real-time operating temperature of the VCSEL. The output voltage and current required when using the LT8722 to control the TEC under varying temperature differences are shown in Fig. 8.

### 3.2. VCSEL temperature without PID compensation

**Fig. 9.**
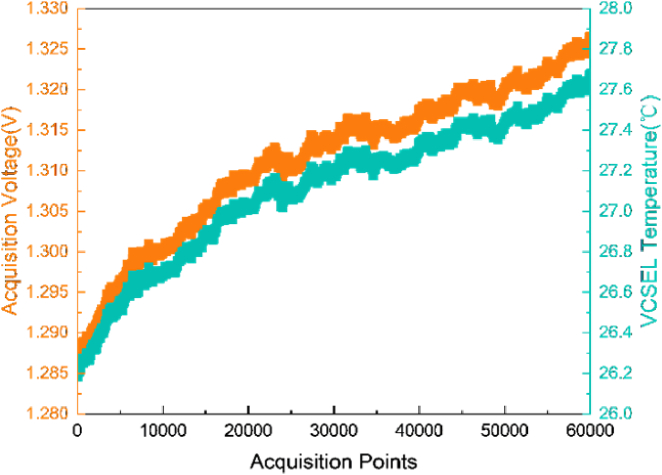
Experimental results showing the acquisition voltage (left y-axis) and real-time VCSEL temperature (right y-axis) over 60,000 sampling points, equivalent to 10 minutes of data.

The performance of the proposed temperature control platform was evaluated by first analyzing the natural temperature drift of the working VCSEL without active temperature control. The experimental setup was maintained at a room temperature of 26.2 °C. The NTC data acquired by the front-end ADC were recorded without applying the PID algorithm. These raw ADC samples were processed by the ZYNQ board and transmitted to a master computer at a sampling rate of 100 Hz. As shown in Fig. 9, an analysis of 60,000 data points, corresponding to 10 minutes of operation, reveals a continuous increase in the operating temperature of the VCSEL in the absence of active temperature control.

**Fig. 10.**
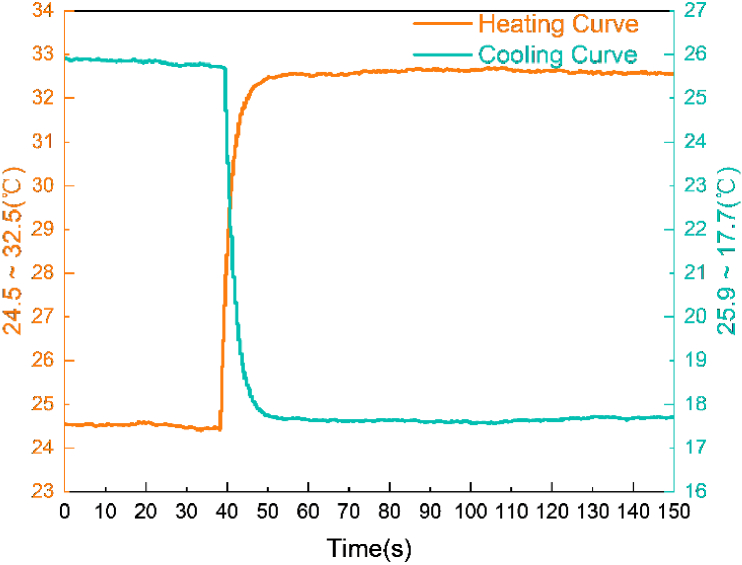
**Temperature control response of the system for VCSEL heating and cooling.** The system adjusts the VCSEL temperature from 24.5°C to 32.5°C for heating and from 25.9°C to 17.7°C for cooling, reaching the target setpoint within approximately 10 seconds.

The capability of this proposed platform to regulate heating and cooling was evaluated by adjusting the VCSEL temperature from 24.5 °C to 32.5 °C for heating and from 25.9 °C to 17.7 °C for cooling. The resulting temperature control curve, shown in Fig. 10, demonstrates that the system can achieve the desired temperature setpoint within approximately 10 seconds.

**Fig. 11.**
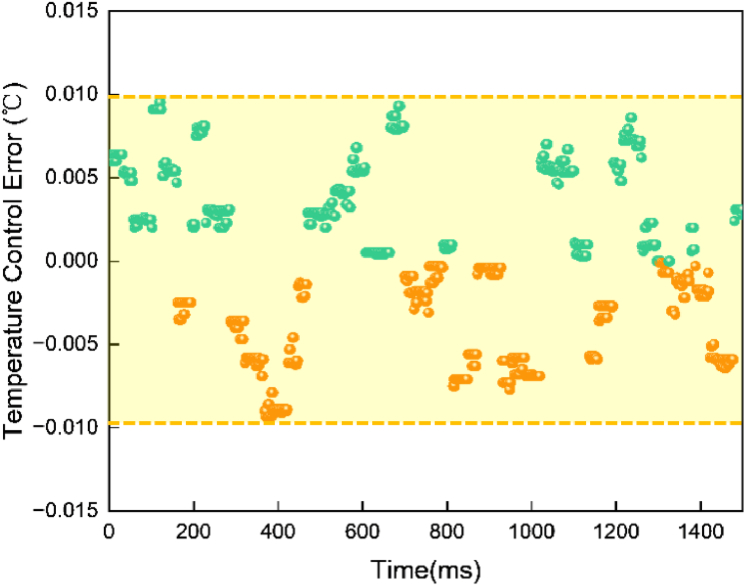
Temperature control error when stabilizing the VCSEL at 25°C.

Figure 11 presents the temperature control error when stabilizing the VCSEL at 25°C. Green markers indicate instances where the measured temperature slightly exceeds the setpoint, while orange markers indicate cases where the measured temperature is slightly below the setpoint. The results show that the system can maintain a stable operating temperature within an error margin of ±0.01 °C. This level of precision meets the stringent requirements of the operation temperature in fNIRS/DOT applications, ensuring reliable and high-resolution neuroimaging measurements.

### 3.3. Resource utilization

**Fig. 12.**
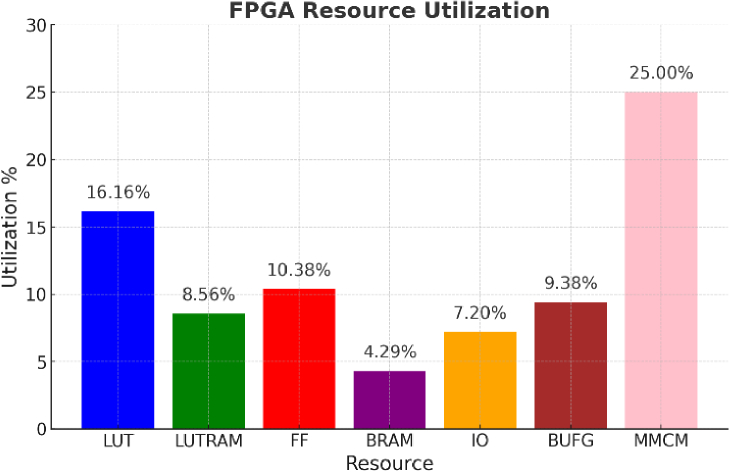
Resource utilization of the proposed ZYNQ-based temperature control system.

The resource utilization of the proposed ZYNQ-based temperature control platform was optimized, ensuring efficient allocation of computational resources. As shown in Fig. 12, the design utilizes 16.16% of the available Look-Up Tables (LUTs), 8.56% of Look-Up Table Random Access Memory (LUTRAM), and 10.38% of flip-flops (FFs), demonstrating a balanced distribution of logic resources. The BRAM utilization remains low at 4.29%, while the Input/Output (IO) and Global Clock Buffers (BUFG) resources are utilized at 7.20% and 9.38%, respectively. The maximum utilization observed is for the Mixed-Mode Clock Manager (MMCM), which accounts for 25% of the available units. These results indicate that the system efficiently manages FPGA resources on the ZYNQ board, leaving a significant portion available for potential future enhancements, such as integrating additional control features or expanding the platform's scalability for (wearable) high-density fNIRS/DOT applications.

To assess the scalability of the proposed architecture, we evaluated the resource implications of integrating additional functional modules, including optical signal acquisition and basic preprocessing. The system employs a time-multiplexed control strategy, with a single instance of the PID computation logic shared across all 128 channels. Each channel maintains its own control state—comprising temperature error, integral value, and control output—in dedicated memory. This design enables dynamic and independent control per channel while avoiding linear growth in logic resource usage. The additional overhead for channel indexing and SPI arbitration is minimal.

Optical signal acquisition components, such as photodetectors and analog front-end circuits, are implemented externally and do not occupy FPGA resources. Within the programmable logic, only simple modules are required for ADC interfacing, buffering, and data forwarding. Preprocessing functions, including moving average filtering and baseline correction, can be implemented using a small number of DSP slices and LUTs. These operations, when executed under the same time-multiplexed scheme, introduce negligible resource overhead.

The dual-core ARM processor integrated in the ZYNQ-7000 handles configuration and sequential control tasks in software. No additional scheduling logic is required in the programmable fabric. Considering all functional blocks, the estimated total resource usage—covering temperature control, signal acquisition, and basic preprocessing—remains below 50%, providing sufficient margin for future expansion.

## 4. Discussion and conclusion

This study presents a high-precision, ZYNQ-based temperature control platform for VCSELs, leveraging the computational efficiency of the heterogeneous ZYNQ-7000 FPGA. The integration of a hardware-accelerated PID controller in the PL allows for real-time temperature regulation, achieving an accuracy of ±0.01 °C. This level of precision is crucial for mitigating wavelength drift and power fluctuations, ensuring stable optical performance of the VCSELs in CW, FD and TD fNIRS/DOT applications.

A key contribution of this work is the scalable multi-channel control architecture, which efficiently controls the temperature of 128 VCSELs while maintaining low resource utilization. The parallel processing capabilities of this platform allow for real-time control without compromising computational efficiency, making it a promising solution for high-density light source configurations. This scalability ensures that VCSELs can fully exploit their superior wavelength tuning precision and modulation capabilities, reinforcing their suitability for next-generation high-density optical neuroimaging technologies. The compact form factor and real-time control capabilities of the proposed system make it well-suited for wearable fNIRS/DOT applications, crucial for diverse applications such as clinical monitoring and brain-computer interfaces.

Furthermore, the low utilization of FPGA resources not only supports the control of a large number of VCSELs but also enables seamless integration with additional system functions. The available resources can be allocated for raw data processing, real-time monitoring, or advanced control strategies, such as adaptive tuning of PID parameters through machine learning algorithms. This adaptability makes the proposed platform highly flexible for broader fNIRS/DOT applications.

Future work will focus on further optimizing resource allocation and expanding system functionality to include real-time data acquisition from light detectors. Additionally, the potential integration of deep learning algorithms on the ZYNQ platform could enhance adaptive temperature control, improving response time and robustness in dynamic environments [21,30,31]. These advancements would further solidify the vital role of this platform in next-generation biomedical imaging and optical sensing applications [32–35].

In conclusion, this work demonstrates a robust and scalable reconfigurable hardware-based temperature control platform that addresses the critical challenge of thermal stability of VCSELs in high-density fNIRS/DOT applications, particularly for wearable fNIRS/DOT technologies. Compared to conventional control approaches, the proposed platform achieves superior precision, fast response times, and greater scalability while maintaining high flexibility for future implementations of extra functions. The proposed platform not only enhances the thermal reliability of wearable high-density fNIRS/DOT technologies but also provides a foundational thermal control framework for even broader applications requiring stable and reliable performance of high-density light sources.

## Data Availability

Data underlying the results presented in this paper are not publicly available at this time but may be obtained from the authors upon reasonable request.
